# Spontaneous malignant glaucoma in a patient with patent peripheral iridotomy

**DOI:** 10.1186/1471-2415-12-64

**Published:** 2012-12-14

**Authors:** Mallika Premsenthil, Mohamad Aziz Salowi, Chong Min Siew, Intan ak Gudom, Tan Kah

**Affiliations:** 1 Department of Ophthalmology, Faculty of Medicine and Health Sciences, Universiti Malaysia Sarawak (UNIMAS), Lot 77, Seksyen 22, Kuching Town Land District, Jalan Tun Ahmad Zaidi Adruce, 93150 Kuching, Sarawak, Malaysia; 2 Department of Ophthalmology, Sarawak General Hospital, 93150 Kuching, Sarawak, Malaysia

## Abstract

**Background:**

To report a case of spontaneous malignant glaucoma in an Asian female. To propose the term “positive vitreous pressure glaucoma” to reflect the pathophysiology, treatment and prognosis of the condition.

**Case presentation:**

A 56-year old Chinese female was diagnosed of primary angle closure glaucoma and had bilateral laser peripheral iridotomy one year ago. She presented with spontaneous onset of malignant glaucoma involving the left eye. The condition was treated successfully; the final best corrected visual acuity was 0.67 (decimal notation).

**Conclusion:**

This case highlights that acute angle closure attack can occur in an eye with patent peripheral iridotomy. Early recognition and treatment is essential for good visual prognosis.

## Background

Malignant glaucoma is a form of secondary angle closure glaucoma characterized by marked elevation of the intraocular pressure (IOP), shallow anterior chamber despite a patent laser peripheral iridotomy (LPI), and a normal posterior segment anatomy. A recent review of modern literature classified malignant glaucomas into classic malignant glaucoma, nonphakic malignant glaucoma and other malignant glaucoma syndromes [1]. Classic malignant glaucoma typically develops in patients with primary angle closure glaucoma after incisional surgery. Non-phakic malignant glaucoma occurs after cataract extraction. Other malignant glaucoma syndromes may be spontaneous, or associated with any ocular pathologies or the use of miotics. We report a case of malignant glaucoma that occurred spontaneously in an eye that underwent LPI one year ago.

## Case presentation

**Figure 1:**
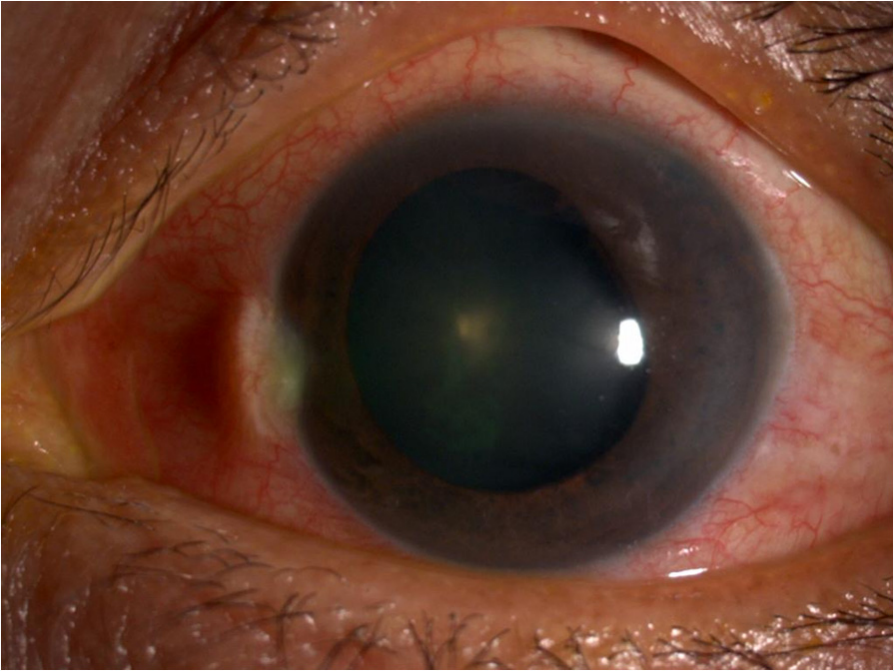
Diffuse illumination, the pupil was mid-dilated pupil with ciliary flush.

**Figure 2:**
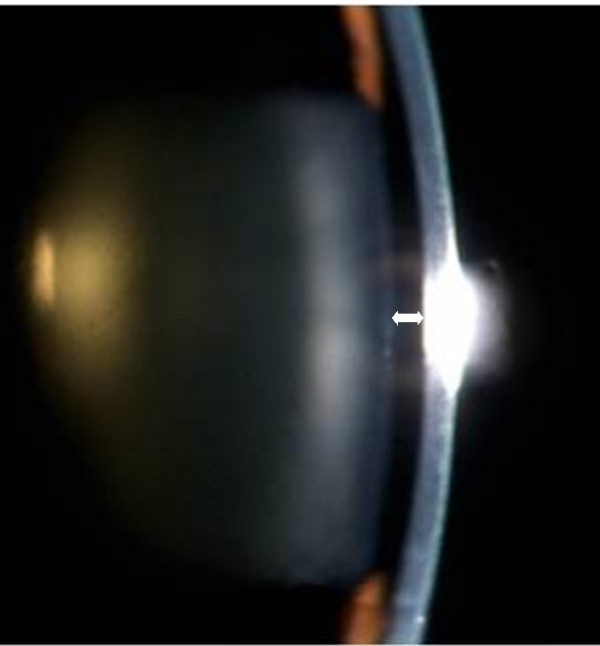
Parallel-piped illumination, the anterior chamber depth was very shallow (double-headed arrow).

A 56-year-old Chinese female with primary angle closure glaucoma, underwent bilateral LPI one year ago. Her eyes were treated with topical timolol 0.5% twice daily and topical latanoprost 0.005% at night. During her last clinic visit, the IOPs were 20 mm Hg (right) and 21 mm Hg (left). She presented with three days’ history of sudden left eye pain, redness, lacrimation and blurring of vision associated with headache. The episode occurred spontaneously. Visual acuity was 0.17 (decimal notation). Ocular examination showed marked ciliary flush, corneal edema and mid-dilated pupil (Figure 1). The anterior chamber was very shallow (Figure 2).

**Figure 3:**
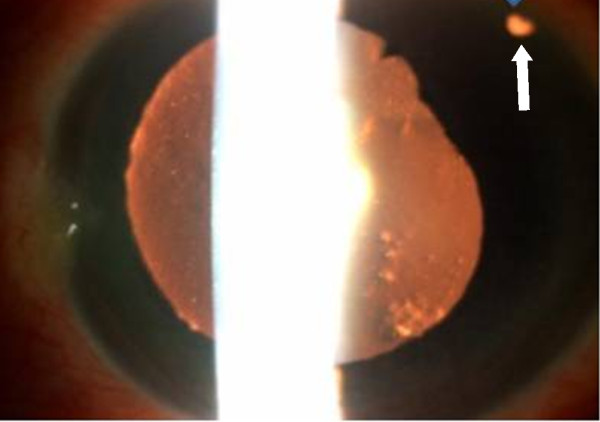
Retro-illumination, patent peripheral iridotomy (white arrow).

**Figure 4:**
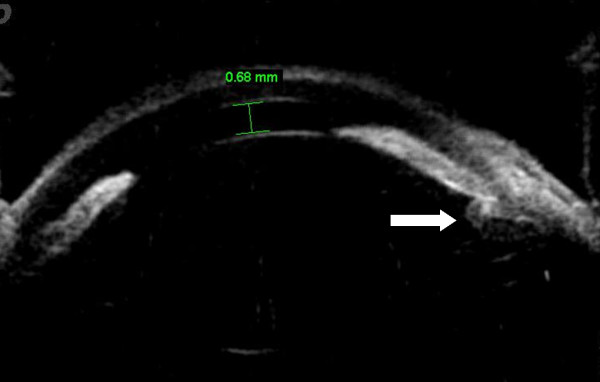
Ultrasonographic biomicroscopy, the central anterior chamber depth was 0.68 mm with anterior rotation of the ciliary body (white arrow) and forward displacement of the iris.

Retro-illumination revealed a patent peripheral iridotomy (Figure 3). The IOP was 60 mm Hg. Relative afferent pupillary defect was absent. The crystalline lens was cataractous with grade 2 nuclear sclerosis. Ultrasonographic biomicroscopy showed peripheral iridocorneal touch and forward rotation of the ciliary body (Figure 4). B-mode ultrasonography showed a normal posterior segment.

The diagnosis of malignant glaucoma was made. She was treated immediately with intravenous mannitol 20%, oral acetazolamide 250 mg, topical atropine 1%, topical timolol 0.5% and topical latanoprost 0.005%. The IOP came down to 26 mm Hg after 2 hour, but subsequently rose to 36 mm Hg with persistent shallowing of the anterior chamber. An emergency anterior vitrectomy was performed via the pars plana, followed by phacoemulsification cataract extraction, primary posterior capsulotomy, and posterior chamber intraocular lens implantation.

**Figure 5:**
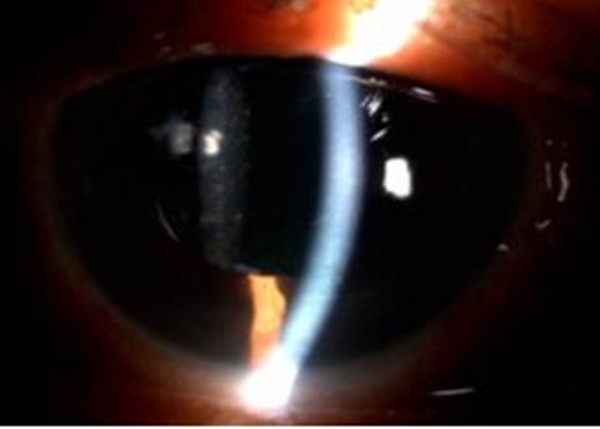
Post-operative photograph of the anterior segment.

**Figure 6:**
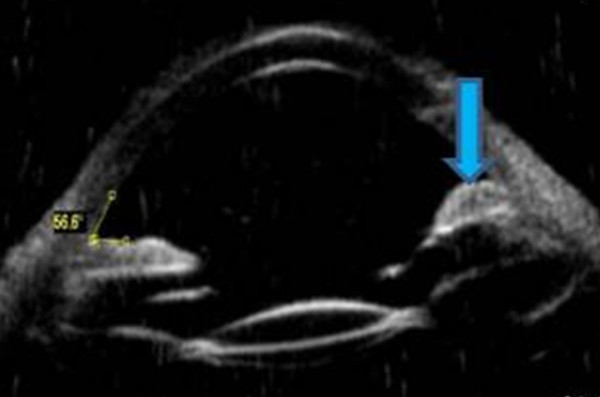
Ultrasonographic biomicroscopy showing backward displacement of the iris (blue arrow).

Postoperatively the anterior chamber depth increased (Figure 5) and the IOP came down to 20 mm Hg. Ultrasonographic biomicroscopy on the fifth postoperative day showed backward displacement of the ciliary body and iris with opened drainage angle (Figure 6). She was discharged after a week with topical atropine 1% daily, topical timolol 0.5% twice daily and topical latanoprost 0.005% at night for both eyes. After 6 month, her best corrected visual acuity was 0.67, IOP was 18 mm Hg.

## Discussion

Primary angle closure is common among Asians. In Singapore, the incidence of acute primary angle closure glaucoma in those aged 30 years and older was reported to be as high as 12.2 per 100 000 per year [2]. However, malignant glaucoma is not commonly reported in Asian population [1]. In a prospective randomized controlled trial in Chinese patients with chronic angle closure glaucoma, none of the patients developed malignant glaucoma as a result of intraocular surgeries [3].

Spontaneous malignant glaucomas are very rare. To the best of our knowledge, there are only two cases of spontaneous malignant glaucoma reported in the literature; both involved Caucasians. The two cases were reported in eyes without antecedent eye surgery or miotics [4,5]. This is the first reported case of spontaneous malignant glaucoma occurring in an Asian patient. Although LPI is known to trigger malignant glaucoma, the attack usually occurs within the period where inflammatory responses due to the procedure are still active [6-9]. A period of one year after the initial LPI makes the association between the procedure and malignant glaucoma unlikely; hence we diagnosed and successfully managed this episode as a spontaneous malignant glaucoma.

The exact pathogenesis of malignant glaucoma remains unclear. Several theories have been proposed as the precipitating event. Chandler believed that laxity of the lens zonule may be responsible for the condition [10]. Later, the posterior aqueous misdirection theory was proposed by Shaffer and Hoskin [11]. Recently, the choroidal expansion theory was described by Quigley et al. [12]. A patent LPI relieves pupillary block, where the differential pressure between the anterior chamber and the posterior chamber is nullified. In the presence of an intact anterior hyaloid face, the vitreous fluid conductivity remains poor. Therefore, a patent LPI does not relieve the pressure differential between the vitreous cavity and the anterior segment. Quigley et al. further pointed out that eyes with primary angle-closure glaucoma have persistent “positive pressure” phenomenon despite patent iridotomy. This is due to the higher-than-normal tendency for choroidal expansion and poor vitreous fluid conductivity. In malignant glaucoma, a vicious cycle of poorer vitreous fluid conductivity and increased transvitreal pressure is established. This results in compression of the vitreous gel, progressive forward displacement of the lens-iris diaphragm and eventual direct closure of the anterior chamber angle despite the presence of patent iridotomy [12].

The term “direct lens block glaucoma” was proposed to differentiate this from pupillary block angle closure [10]. However, aphakic malignant glaucoma has been reported in patient underwent intracapsular cataract extraction [13]. Shahid and Salmon suggested that the term “malignant glaucoma” is no longer suitable as the prognosis is good with the current treatment modalities [1]. The definitive management of malignant glaucoma is the removal of the posterior capsule and the anterior vitreous. Free fluid movement between the anterior and posterior segment of the eye relieves the positive vitreous pressure and prevent future recurrence [14].

We proposed the term “positive vitreous pressure glaucoma” to replace “malignant glaucoma” as:

i. the phrase “positive vitreous pressure” reflects the final common pathway leading to the development of vicious cycle of increased in transvitreal pressure,

ii. treatments should address the 2 important contributing factors for the development of this condition; reduction of choroidal expansion and facilitation of vitreous fluid conductivity [12], and

iii. the term “malignant” is no longer suitable to reflect the prognosis of the condition [1].

## Conclusion

Malignant glaucoma is a rare but sight threatening condition. This case highlights that acute angle closure attack can occur in an eye with patent peripheral iridotomy. Early recognition and treatment is essential as the prognosis of this condition is favorable with the current treatment modalities. The term “positive vitreous pressure glaucoma” is proposed to reflect the pathophysiology, treatment and prognosis of the condition.

### Consent

Written informed consent was obtained from the patient for publication of this case report and any accompanying images. A copy of the written consent is available for review by the Editor-in-Chief of this journal.

## Competing interests

The authors declare that they have no competing interests.

## Authors’ contributions

MP, MAS, CMS and IG treated the patient and in doing so acquired the case data; all were also involved with drafting of the manuscript. TAK coined the term “positive vitreous pressure glaucoma”. All authors read and approved the final manuscript.

## Pre-publication history

The pre-publication history for this paper can be accessed here:

http://www.biomedcentral.com/1471-2415/12/64/prepub
